# Prevalence of hepatitis-C virus genotypes and potential transmission risks in Malakand Khyber Pakhtunkhwa, Pakistan

**DOI:** 10.1186/s12985-017-0829-y

**Published:** 2017-08-22

**Authors:** Nausheen Nazir, Muhammad Rasul Jan, Amjad Ali, Muhammad Asif, Muhammad Idrees, Mohammad Nisar, Muhammad Zahoor, Naser M. Abd El-Salam

**Affiliations:** 1 Department of Botany, University of Malakand, Chakdara Dir (L), Khyber Pakhtunkhwa, Pakistan; 2 Institute of Chemical Sciences, University of Peshawar, Peshawar, Khyber Pakhtunkhwa Pakistan; 3 Centre for Applied Molecular Biology, University of the Punjab, 87-West Canal Bank Road Thokar Niaz Baig, Lahore, Pakistan; 4 Department of Statistics, University of Malakand, Chakdara Dir (L), Khyber Pakhtunkhwa, Pakistan; 5 Vice Chancellor Hazara University, Mansehra, Pakistan; 6 Department of Chemistry, University of Malakand, Khyber Pakhtunkhwa, Chakdara Dir (L), Pakistan; 7 Riyadh Community College, King Saud University, Riyadh, 11437 Saudi Arabia

**Keywords:** HCV, Genotypes potential, Risks factors, Distribution patterns, Malakand

## Abstract

**Background:**

Hepatitis C virus (HCV) is a leading cause of chronic liver disease and frequently progresses towards liver cirrhosis and Hepatocellular Carcinoma (HCC). This study aimed to determine the prevalence of HCV genotypes and their association with possible transmission risks in the general population of Malakand Division.

**Methodology:**

Sum of 570 serum samples were collected during March 2011 to January 2012 from suspected patients visited to different hospitals of Malakand. The suspected sera were tested using qualitative PCR and were then subjected to molecular genotype specific assay. Quantitative PCR was also performed for determination of pre-treatment viral load in confirmed positive patients.

**Results:**

Out of 570 serum samples 316 sera were seen positive while 254 sera were found negative using qualitative PCR. The positive samples were then subjected to genotyping assay out of 316, type-specific PCR fragments were seen in 271 sera while 45 samples were found untypable genotypes. Genotype 3a was seen as a predominant genotype (63.3%) with a standard error of ±2.7%. Cramer’s V statistic and Liklihood-Ratio statistical procedures are used to measure the strength and to test the association, respectively, between the dependent variable, genotype, and explanatory variables (e.g. gender, risk, age and area/districts). The dependent variable, genotype, is observed statistically significant association with variable risk factors. This implies that the genotype is highly dependent on how the patient was infected. In contrast, the other covariates, for example, gender, age, and district (area) no statistical significant association are observed. The association between gender-age indicates that the mean age of female was older by 10.5 ± 2.3 years with 95% confidence level using *t-statistic*.

**Conclusion:**

It was concluded from the present study that the predominant genotype was 3a in the infected population of Malakand. This study also highlights the high prevalence rate of untypable genotypes which an important issue of health care setup in Malakand and create complications in therapy of infected patients. Major mode of HCV transmission is multiple uses and re-uses of needles/injections.

**Trial registration:**

ISRCTN ISRCTN73824458. Registered: 28 September 2014

## Background

Hepatitis C (HCV) is an enveloped RNA virus that was firstly discovered in 1989 having 9.6 Kb genome flanked at both ends by untranslated regions (5 ‘UTR and 3’ UTR). The HCV genome encodes 3008–3037 amino acids of single polyprotein and processed post translationally produce three distinct structural proteins and six non-structural proteins [1].

An estimated 70% to 85% of HCV patients are likely to develop chronic hepatitis, and up to 30% of these cases progress towards liver cirrhosis [2]. In year 2013, hepatitis C was the foremost cause of 1.46 million deaths worldwide and 7.2 million deaths were expected from the years 2015–2030 [3]. An estimated 130–170 million world’s population is chronically infected with hepatitis C, while highest prevalence was observed in Asia and Africa [4]. The distribution pattern of hepatitis C is inconsistent from 4%–12% in Asia-pacific regions [5, 6]. About 10 million people have been infected with HCV in Pakistan [7] and the prevalence rate is 4% [8]. In China, the prevalence rate ranged from 1% to 31.86% depends on its regions of lowest and highest endemic cities [9] while the prevalence rate is 1.8% in Saudi Arabia [10].

World Health Organization (WHO) organized the first Global Health Sector Strategy (GHSS), 2016–2021, for the prevention and control of viral hepatitis. GHSS would try to stop the expected 7.1 million HCV linked deaths during years 2015–2030 and would achieve the health targets of the 2030 Agenda for Sustainable Development to combat viral hepatitis [11].

HCV is attributed 27% cirrhosis and 25% HCC globally [12] and is the major cause of liver transplantation [13].

HCV is classified into six major genotypes on the basis of nucleotide heterogeneity. Genotypes 1 and 3 are circulating predominantly across the globe. In Pakistan genotype 3 is the predominant genotype with subtype 3a and 3b circulating with same pattern in males and females [14–17]. Genotype 4 is more prevalent in North Africa and Middle East while in the Hong Kong and South Africa genotypes 5 and 6 are circulating [14].

The common route of HCV transmission in developing country is the re-uses of needles/syringes and unsafe injections [17, 18]. The improper sterilized medical apparatus, unsafe blood transfusion and re-uses of needles/syringes and unsafe injections causes an estimated of 2 to 5 million HCV infections [19]. The observed risks factors for HCV transmission in Pakistan were including uses & re-uses of needles/injections unsafe injections, dental procedures, surgeries (major/minor), blood transfusion, barbers, piercing instruments and about 1% due to vertical transmission [20, 21]. The reported literatures from Pakistan have shown the prevalence of HCV-3a infections in patients who have received multiple unsafe injections by untrained health practitioners mainly in rural areas [21–25].

In Pakistan many reported studies are available on the prevalence of HCV genotypes and their possible routes of transmission in various districts/cities [7, 14–17, 21, 22, 25–27]. However no such type of study is documented on the prevalence of HCV genotypes and their association with covariates i.e. age, gender, possible routes of transmission in Malakand Division, Khyber Pakhtunkhwa (KP). So this study aimed to determine the prevalence of different HCV genotypes and their possible routes of transmission in different districts of Malakand, KP, Pakistan.

## Materials and methods

### Blood sampling

Sum of 570 blood samples were collected from suspected patients visited to different hospitals of Malakand KP. Informed consent was taken in written form from each patient including, demographic characteristic, age, district, risk factor and estimated time of infection along with complete address and phone numbers.

### HCV RNA qualitative and quantitative PCRs

Blood samples were used for qualitative analysis of HCV-RNA as described previously [28]. Total RNA from the suspected patient’s sera (100 μl) was extracted using Quigen RNA extraction kit. RT-PCR was used for the detection of HCV-RNA. 20 μl reaction mix was used for Nested PCR using Taq DNA-polymerase (Fermentas, Technologies, USA) and products were visualized under UV light using “Uvitec” gel documentation system on 2% agarose gel.

HCV RNA was quantified in all qualitative PCR positive sera using Smart Cycler-II Real-time PCR (Cepheid, Calif, and Sunnyvale, USA) using HCV-RNA quantification kits (Sacace, Biotechnologies, Italy). The Smart Cycler-II PCR undergoes amplification and identification at the same instant with Taq-Man technology (Applied Biosystems, Calif, Foster City) through fluorescent probes following each replicating cycle. The lower detection limit was 250 IU/mL and upper detection limit was 5.0 × 108 IU/mL, respectively. Samples yielding values above the upper limit were diluted 100-fold, retested and the obtained values were multiplied by this dilution factor to get the actual HCV RNA concentration in international units (IU) per mL.

### HCV genotyping

The qualitative PCR positive sera were subjected to HCV genotyping by using type-specific HCV genotyping procedure as described previously [26]. Briefly, 10 μl (50 ng) HCV-RNA was reverse transcribed into cDNA at 37 °C for 50 min. Two μl of synthesized cDNA was utilized for PCR amplification of 470-bp region from HCV 5′NCR along with core region by 1st round PCR amplification. The amplified 1st round PCR product was subjected to two 2nd rounds of nested PCR amplifications. Two reaction mixes were made, 1st reaction with mix-A primers set and the 2nd reaction with mix-B primers set in a reaction volume of 10 μl. Mix-A had specific genotype primers set for 1a, 1b, 1c, 3a, 3c and 4 genotypes and mix-B contained specific genotype primers set for 2a, 2c, 3b, 5a and 6a.

### Statistical analysis

SPSS version 17.0 for Windows was used for the analysis of data and summary statistics. The results for all variables were set in the form of rates (%). T-test is used to test the equality of the two proportions, whereas, the Chi Square tests is used to test the equality of more than two proportions. Further, Chi-Square test, Log-Likelihood Ratio test (LR) and Cramer’s V statistics were applied to test out the significance of association among the categorical variables [29, 30]. The level of significance is set as 0.05, therefore, if any *p*-value observed less than 0.05 was considered as statistical significance.

## Results

Out of 570 blood samples 316 samples were seen positive while 254 found negative using qualitative PCR. The qualitative PCR positive samples were subjected to genotyping assay out of 316 type-specific PCR fragments were seen in 271 serum samples whereas 45 samples were found with untypable genotypes as no genotype-specific band was seen in these samples.

**Fig. 1:**
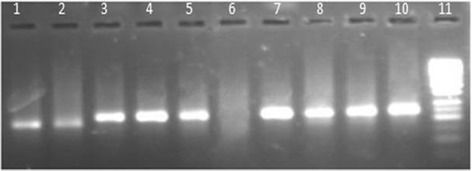
Agarose gel (2%) electrophoresis illustrates results for genotyping of HCV specimens by multiplex PCR as prescribed previously [26]. Lanes 1, 2 showing genotype 1a (129-bp); Lanes 3, 4, 5 &7–10 showing genotype 3a (258-bp); Lane 6 shows negative control and Lane 11 contain 50-bp DNA ladder marker

Distribution breakup of HCV infected population was 49 (15.5%), 96 (30.4%), 61(19.3%), 52 (16.5%) and 58(18.4%) from Batkhela, Swat, Bunir, Shangla and Dir (lower) districts respectively. Fig. 1 shows a typical agarose gel showing different HCV genotype-specific bands (HCV-1a & HCV-3a).

### Gender wise distribution of HCV genotypes among the studied patients

Table 1 demonstrates the cross-tabulation of HCV genotype and gender. Out of 316 HCV patients, males subjects were 171(54.1%) and females were 145(45.9%). Each cell of the table contained the actual frequency, the percentage prevalence within the genotypes and gender. In this study the genotype “3a” is observed most frequently in both male and female patients. Moreover, no significant changes were observed by comparing the proportions of male and female for each category of the genotype, as all the *p* values were greater than 0.05 (Table 1). For this purpose we used the t-test to test the hypothesis of the female percentage equal to 50%.

**Table 1:** Gender wise distribution of HCV genotypes among the studied patients

			Genotype						Total
Gender			1a	1b	3a	3b	Mixed	Untypable	
	Female	Count	4	6	91	11	10	23	145
		% prevalence within Gender	2.8%	4.1%	62.8%	7.6%	6.9%	15.9%	100.0%
		% prevalence within Genotype	26.7%	66.7%	45.5%	44.0%	45.5%	51.1%	45.9%
	Male	Count	11	3	109	14	12	22	171
		% prevalence within Gender	6.4%	1.8%	63.7%	8.2%	7.0%	12.9%	100.0%
		% prevalence within Genotype	73.3%	33.3%	54.5%	56.0%	54.5%	48.9%	54.1%
Total		Count	15	9	200	25	22	45	316
		% prevalence within Gender	4.7%	2.8%	63.3%	7.9%	7.0%	14.2%	100.0%
		*p*-value	0.118	0.508	0.229	0.69	0.83	0.99	100.0%

Gender * Genotype Cross tabulation, The *p*-values are for comparing proportion of Female with 50%

### Distribution of HCV genotypes in different districts of Malakand

The cross-tabulation of the two categorical variables, genotype and district are provided in the Table 2 in which no statistical evidence is observed to confirm the strong association.

**Table 2:** Prevalence of HCV genotypes in different geographical regions of Malakand

			Genotype						Total
			1a	1b	3a	3b	Mixed	Untypable	
District	Batkhela	Count	2	1	34	5	2	5	49
		% prevalence within District	4.1%	2.0%	69.4%	10.2%	4.1%	10.2%	100.0%
		% prevalence within Genotype	13.3%	11.1%	17.0%	20.0%	9.1%	11.1%	15.5%
	Bunir	Count	0	0	42	2	4	13	61
		% prevalence within District	.0%	.0%	68.9%	3.3%	6.6%	21.3%	100.0%
		% prevalence within Genotype	.0%	.0%	21.0%	8.0%	18.2%	28.9%	19.3%
	Dir(Lower)	Count	4	3	34	7	5	5	58
		% prevalence within District	6.9%	5.2%	58.6%	12.1%	8.6%	8.6%	100.0%
		% prevalence within Genotype	26.7%	33.3%	17.0%	28.0%	22.7%	11.1%	18.4%
	Shangla	Count	3	4	31	5	3	6	52
		% prevalence within District	5.8%	7.7%	59.6%	9.6%	5.8%	11.5%	100.0%
		% prevalence within Genotype	20.0%	44.4%	15.5%	20.0%	13.6%	13.3%	16.5%
	Swat	Count	6	1	59	6	8	16	96
		% prevalence within District	6.3%	1.0%	61.5%	6.3%	8.3%	16.7%	100.0%
		% prevalence within Genotype	40.0%	11.1%	29.5%	24.0%	36.4%	35.6%	30.4%
Total		Count	15	9	200	25	22	45	316
		% within District	4.7%	2.8%	63.3%	7.9%	7.0%	14.2%	100.0%
		% within Genotype	100.0%	100.0%	100.0%	100.0%	100.0%	100.0%	100.0%

In contrast, we are able to compare the prevalence of given genotype, i.e. “3a”, within a district with that of the prevalence of the same genotype within another district. For example, it can be observed that the frequency distribution of genotype within each district is with highest frequency percentage of the common type “3a” in all districts. It can be seen from Table 2, the percentages of “3a” for district Batkhela, Bunir, Dir (L), Shangla, and Swat are 69.4%, 68.9%, 58.6%, 59.6% and 61.5% respectively.

### Distribution of HCV genotypes in different age groups

The count summaries of HCV genotypes in various age groups are provided in the Table 3. No statistical significance difference is observed between HCV genotypes and different age groups. The *p*-value to test the significance of the association is observed as 0.484 using the LR test. The detailed summary of the tests is provided in the Table 4. Similar to other categorical variable, the cross tabulation of genotype and age group was also performed. In contrast, the prevalence of the given genotype within age group can possibly be compared with that of the prevalence of the same genotype within another age group. For example, it can be observed from Table 3 that the distributions of genotype within each age group with common genotype 3a. For example, the percentages of the 3a genotype within age-group for age categories 10–20, 20–30, 30–40, 40–50, 50–60, and 60+ are 56.5%, 65.4%, 63.9%, 61.5%, 60.0, and 100%. Regarding the highest percentage of the 60+ category is due to limited data as only five patients are with the age more than 60 years that all have infected with 3a HCV genotype.

**Table 3:** Prevalence of HCV genotypes in different age groups of HCV patients

			Genotype						Total
			1a	1b	3a	3b	Mixed	Untypable	
Age group	10–20	Count	0	0	13	2	0	8	23
		% prevalence within Age group	.0%	.0%	56.5%	8.7%	.0%	34.8%	100.0%
		% prevalence within Genotype	.0%	.0%	6.5%	8.0%	.0%	17.8%	7.3%
	20–30	Count	4	1	53	5	7	11	81
		% prevalence within Age group	4.9%	1.2%	65.4%	6.2%	8.6%	13.6%	100.0%
		% prevalence within Genotype	26.7%	11.1%	26.5%	20.0%	31.8%	24.4%	25.6%
	30–40	Count	7	3	62	8	5	12	97
		% prevalence within Age group	7.2%	3.1%	63.9%	8.2%	5.2%	12.4%	100.0%
		% prevalence within Genotype	46.7%	33.3%	31.0%	32.0%	22.7%	26.7%	30.7%
	40–50	Count	4	4	40	5	6	6	65
		% prevalence within Age group	6.2%	6.2%	61.5%	7.7%	9.2%	9.2%	100.0%
		% prevalence within Genotype	26.7%	44.4%	20.0%	20.0%	27.3%	13.3%	20.6%
	50–60	Count	0	1	27	5	4	8	45
		% prevalence within Age group	.0%	2.2%	60.0%	11.1%	8.9%	17.8%	100.0%
		% prevalence within Genotype	.0%	11.1%	13.5%	20.0%	18.2%	17.8%	14.2%
	60+	Count	0	0	5	0	0	0	5
		% prevalence within Age group	.0%	.0%	100.0%	.0%	.0%	.0%	100.0%
		% prevalence within Genotype	.0%	.0%	2.5%	.0%	.0%	.0%	1.6%
Total		Count	15	9	200	25	22	45	316
		% prevalence within Age group	4.7%	2.8%	63.3%	7.9%	7.0%	14.2%	100.0%
		% prevalence within Genotype	100.0%	100.0%	100.0%	100.0%	100.0%	100.0%	100.0%

**Table 4:** Summary of statistical tests/strength of association among genotypes vs. gender, risk factor, district and age group in 316 HCV suspected patients

	Method			
	Likelihood-Ratio test		Strength of Association	
	LR-Statistic	*p*-value	Cramer’s V	*p*-value
Genotype * Gender	4.462 (5)	0.485	0.117	0.501
Genotype * Risk factor	**64.1 (20)**	**0.000**	**0.225**	0.000*
Genotype * District/area	26.61 (20)	0.147	0.135	0.290
Genotype * Age group	29.25 (25)	0.254	0.125	0.484

Degree of freedom are mentioned in the bracket along with the test statistic

* Highly significant

### HCV genotypes and its association with different risk factors

**Fig. 2:**
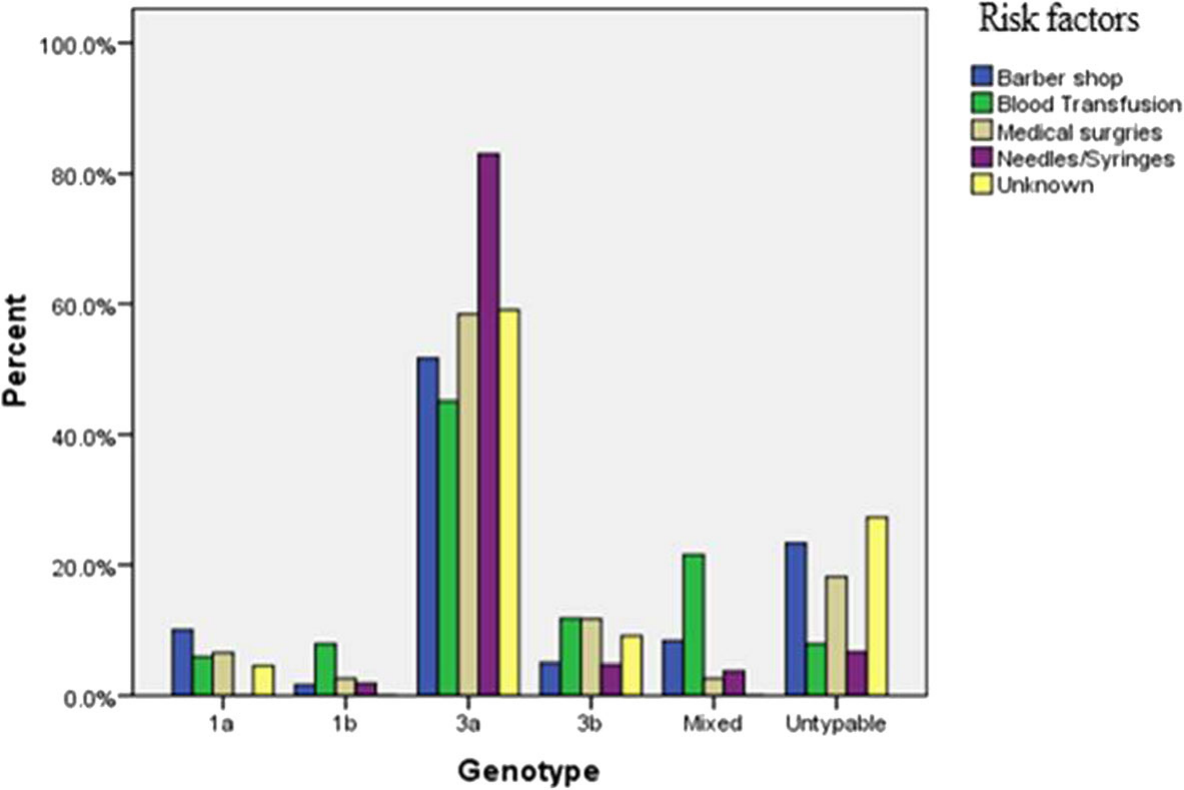
Risk factors assessment of genotypes for HCV infection

The possible risk factors correlated with HCV genotypes are represented in Table 5 & Fig. 2. The potential risk factors are Barber Shop 60(19.0%), Blood Transfusion 51(16.1%), Medical Surgeries 77(20.3%), uses and re-uses of needles/syringes 106(33.5%), and others were unknown 22(7.0%). The LR test suggests that there is strong evidence in the sample data that there exist statistically significant association between variable of interest, genotype and risk-factor. The *p*-value as described in the Table 4 is observed as smaller than our pre-defined level of significance, 0.05. Table 6 describes the count summary of the sample data, the proportion along with the standard error of the proportion. Further, the mean age along with standard error of each category are also provided. Regarding the distribution of the genotype, the variable of interest in this study, it can be observed that the most common genotype in the sample is 3a accounted 63.3% patients with standard error 2.71%. It can also be noticed that there were (14.2±1.96) % patients whom genotype was untypable. ﻿

**Table 5:** Risk factors assessment for HCV infection

			Genotype						Total
Risk factors			1a	1b	3a	3b	Mixed	Untypable	
	Barbers	Count	6	1	31	3	5	14	60
		% prevalence within Risk factor	10.0%	1.7%	51.7%	5.0%	8.3%	23.3%	100.0%
		% prevalence within Genotype	40.0%	11.1%	15.5%	12.0%	22.7%	31.1%	19.0%
	Blood transfusion	Count	3	4	23	6	11	4	51
		% prevalence within Risk factor	5.9%	7.8%	45.1%	11.8%	21.6%	7.8%	100.0%
		% prevalence within Genotype	20.0%	44.4%	11.5%	24.0%	50.0%	8.9%	16.1%
	Medical surgeries	Count	5	2	45	9	2	14	77
		% prevalence within Risk factor	6.5%	2.6%	58.4%	11.7%	2.6%	18.2%	100.0%
		% prevalence within Genotype	33.3%	22.2%	22.5%	36.0%	9.1%	31.1%	24.4%
	Needles/syringes	Count	0	2	88	5	4	7	106
		% prevalence within Risk factor	0.0%	1.9%	83.0%	4.7%	3.8%	6.6%	100.0%
		% prevalence within Genotype	0.0%	22.2%	44.0%	20.0%	18.2%	15.6%	33.5%
	Unknown	Count	1	0	14	2	0	5	22
		% prevalence within Risk factor	4.5%	0.0%	63.6%	9.1%	0.0%	22.7%	100.0%
		% prevalence within Genotype	6.7%	0.0%	7.0%	8.0%	0.0%	11.11%	7.0%
		Total count	15	9	200	25	22	45	316
		% prevalence within Risk factor	4.7%	2.8%	63.3%	7.9%	7.0%	14.2%	100.0%
		*p*-value for within Genotype risk comparison	N/A^a^	N/A^a^	0.000	0.199	0.042^**b**^	0.044	100.0%

^a^ Insufficient data, therefore, Chi-Square test of equal proportion cannot be applied

^b^ The category ‘Unkown’ is ignored while the calculation of the Chi-Square statistic and corresponding *P*-value

**Table 6:** The case summary of 316 patients on the categorical variables information, reported from Malakand division Pakistan

Categorical variables			N	Percent ± S.E	Mean Age ± SE
Variable of Interest	Genotype	1a	15	04.7 ± 1.19	34.20 ± 2.279
		1b	9	02.8 ± 0.93	42.44 ± 3.392
		3a	200	63.3 ± 2.71	37.56 ± 0.906
		3b	25	7.9 ± 1.52	38.88 ± 2.444
		Mixed	22	7.0 ± 1.43	39.55 ± 2.371
		Untypable	45	14.2 ± 1.96	35.42 ± 2.018
Risk factors	Gender	Female	145	45.9 ± 2.80	43.17 ± 1.036
		Male	171	54.1 ± 2.80	32.65 ± 0.797
	Risk factors	Barber shop	60	19.0 ± 2.21	30.23 ± 0.814
		Blood Transfusion	51	16.1 ± 2.07	29.29 ± 1.667
		Medical surgeries	77	24.4 ± 2.42	32.45 ± 1.044
		Needles/Syringes	106	33.5 ± 2.65	44.80 ± 0.810
		Unknown	22	07.0 ± 1.43	58.50 ± 2.343
	District	Batkhela	49	15.5 ± 2.03	25.35 ± 1.078
		Bunir	61	19.3 ± 2.22	38.00 ± 1.146
		Dir(Lower)	58	18.4 ± 2.18	50.17 ± 1.509
		Shangla	52	16.5 ± 2.09	43.33 ± 1.457
		Swat	96	30.4 ± 2.59	32.50 ± 0.944
Total			316		37.48 ± 0.706

The Standard Errors (S.E) of the corresponding sample proportions (expressed in percentages) and mean age are determine by the property of the sampling distribution

In addition to the test results, the cross-tabulation summary is also provided in the Table 5. It can be observed by visual inspection that the distribution of the genotype changes with respect to changes in Risk factor. For example, a total 51 patients were infected because of blood transfusion, out of which 23 (45.1%) were genotyped 3a. In contrast, this percentage increased to 83.3% if the patients are infected due to reuses of needles and unsafe injection.

### HCV RNA viral titers

Pretreatment viral titer was classified of the typable and untypable genotypes into three categories based on its level such as low (< 60, 0000 IU/ml), intermediate (60, 0000–80, 00000 IU/ml) and high (> 80, 00000 IU/ml) viral titer. All the HCV RNA positive samples of current study were genotyped using reported PCR genotyping assay [26]. Base line HCV viral titer for typable genotypes was low (< 60, 0000 IU/ml) and intermediate (60, 0000 to 80, 00000 IU/ml) while the viral titer of untypable genotype was seen high (> 80, 00000 IU/ml), so untypability was not due to low viral titer but due to changes in genotypes sequences.

## Discussion

Malakand division is situated in the Khyber Pukhtunkhwa region of Pakistan lies at a known historic position and acts as a gateway to Chitral, Dir, Baja War and Swat. The present study aimed to determine the distribution of HCV genotypes and their potential transmission risks in Malakand. The data was categorized for analysis in terms of gender, risk factors, age groups and locality. In the present study correlation of HCV genotypes were reported with gender. It was confirmed that there was no variation of HCV genotypes distribution among both sexes all the genotypes were circulated with the same pattern in male and female patients. The same results were also demonstrated by previous reported studies and confirmed that there is no difference in distribution of HCV genotypes between male and female patients [15, 31]. But our results were contradicted from previous reported study that HCV genotypes were not distributed with same pattern where as HCV genotype 1 is circulating in male subjects while genotype 4 in female patients [32].

In our findings the frequently circulated genotype was 3a similar results was also reported by previous studies conducted in different districts/towns of Khyber Pakhtunkhwa and confirmed that the predominant genotype was 3a [15, 16, 21, 27, 33–36]. Our results are also in accordance with another study conducted in Lahore, showed the predominant prevalence of genotype 3a [37]. Another epidemiological study was also conducted in seven different regions of Baluchistan province of Pakistan and reported that 3a was the predominant genotype [38]. Available studies on the distribution of HCV genotypes in different districts/areas of Pakistan have confirmed that predominant genotype was 3a [18, 20–23, 25, 31, 37, 39, 40]. So the present study confirmed that in general population of Malakand HCV type 3a–infected patients are high as compared to other genotypes.

Our results were also similar to our neighboring country like India and in far away Asian country like Nepal and confirmed that most prevalent circulating in these countries was type 3a [41] but dissimilar to Americas, Europe and Japan where 1 and 2 are commonly circulating genotypes [42]. In North Africa and Middle East genotype 4 is prevalent while in Hong Kong and South Africa 5 and 6 genotypes were present [14]. In our study genotypes 4, 5 and 6 were not identified, also confirmed by other studies that these genotypes are not circulating in this region or partially absent from Pakistan [15, 34].

Findings of the this study confirmed that there is no geographical variation among the distribution of HCV genotypes in Malakand all the genotypes were distributed, with same pattern in all districts and similar results were also confirmed by another reported study that all the genotypes were circulating with the same pattern in different regions of Pakistan [15, 40].

An interesting finding of our study is the number of untypable genotypes that produced no genotype-specific PCR fragments in our genotyping assay [26]. All the untypable genotypes had sufficient viral titer indicating that the untypability was not due to low HCV levels. Because untypable HCV genotypes have also been reported in other studies from Pakistan [34–36], this suggests the presence of new genotypes and/or quasispecies which may present a critical health care issue in Pakistan if there are difficulties in treatment of these patients. As such, there is a need to sequence these untypable HCV samples to determine the cause of this problem and possibly to identify appropriate primers for these potential new sub-genotypes to reduce the number of untypable HCV genotypes.

The results were further analyzed for different age groups it was investigated from the overall mean age and *SE* of mean (37.48 ± 0.706) that the high prevalence of HCV infection was seen in age group of ≤40 years. Our findings are in agreement with previous published studies that highest frequency of HCV infection was seen in age group of ≤40 years as compared to ≥40 age group which revealed that the general population of this region is alert for early HCV diagnosis [15, 40].

The study was further analyzed to correlate various HCV genotypes with their potential transmission risks. Statistically significant association is observed between response variable genotype and one covariate risk-factor in the data using the Log likelihood-Ratio test. For example, it can be observed that the highest number of patients, 88 out of 200 infected patients with genotype 3a, was exposed to the multiple uses and reuses of contaminated needles /syringes. However, in contrast no patient was observed with genotype 1a that is infected due to the reuse of the needles/Injections. It was suggested by the previously countrywide reports that 3a is the common genotype among individuals used contaminated needles/syringes [14, 17, 18]. In Pakistan the utilization of injections per person annually ranged from 8.2–13.6 which is maximum range amongst under developing countries [18]. In regards to the overall distribution of the infected patients with respect to the risk-factor, the highest prevalent risk factor is the reuses of needles/injections that possibly cause to infect 106 patients in out of 316. Similarly, 2nd most prevalent risk factor is a medical/dental surgeries (both major and minor) confirmed that medical experts and dentists used unsterilized surgical instruments and consider main contributors in spreading of HCV infection in healthy population [15, 22]. Exposure to barber shops is also a major risk for HCV infection reported previously [35, 43]. In our data we also reported that barbers are also contributes in HCV infection spreading to healthy population because most of the barbers uneducated and reuse contaminated razors/blades for general population of this region/area. Another risk factor is transfusion of blood is accounted 16.1% in majority of HCV thalassaemic patients who had received blood transfusion many times in life and suggests that blood in that area not screen properly before transfusion and is a major issue for thalassaemic patients and general healthy population.

Limitations of this study: first limitation was the detection of large number of HCV untypable genotypes. The detected HCV untypable samples had sufficient viral titers and required to sequence these samples for identification of exact genotype, but we were unable to sequence these untypable samples because the lack of sequencing facilities in our institution another limitation of our study is the risk factor data is self-reported by patients.

## Conclusions

It is concluded from the current study that 3a is the most common genotype. The common transmission route is the reuses of needles/syringes and unsafe injections. Further, the statistical dependence of risk factor on the distribution of genotype is observed. However, in contrast, no statistical dependence on covariates, age, gender and district, is identified. Local variation in the circulation of genotypes was not seen in the current study, all the genotypes/subtypes were present with similar pattern in different district/areas of Malakand. A strong campaign should be needed to inform the health care professionals and dispensers of the rural areas to avoid the reuses of needles /syringes and unsafe injections to control further spreading of HCV.

## Abbreviations

cDNA: Complementra DNA
HCV: Hepatitis C Virus
LR: Log-Likelihood Ratio test
SE: Standard error
UTR: Untranslated Region
